# Idiopathic inflammatory myopathies: pathogenic mechanisms of muscle weakness

**DOI:** 10.1186/2044-5040-3-13

**Published:** 2013-06-07

**Authors:** Sree Rayavarapu, William Coley, Travis B Kinder, Kanneboyina Nagaraju

**Affiliations:** 1 Research Center for Genetic Medicine, Children’s National Medical Center, 111 Michigan Ave NW, Washington DC, USA; 2 Institute of Biomedical Sciences, The George Washington University, 2300 Eye Street, N.W., Ross 605, Washington DC, USA

**Keywords:** Adaptive immune, Autophagy, Cytokines, Endoplasmic reticulum stress, Innate immune, Myositis, Skeletal muscle, TLRs

## Abstract

Idiopathic inflammatory myopathies (IIMs) are a heterogenous group of complex muscle diseases of unknown etiology. These diseases are characterized by progressive muscle weakness and damage, together with involvement of other organ systems. It is generally believed that the autoimmune response (autoreactive lymphocytes and autoantibodies) to skeletal muscle-derived antigens is responsible for the muscle fiber damage and muscle weakness in this group of disorders. Therefore, most of the current therapeutic strategies are directed at either suppressing or modifying immune cell activity. Recent studies have indicated that the underlying mechanisms that mediate muscle damage and dysfunction are multiple and complex. Emerging evidence indicates that not only autoimmune responses but also innate immune and non-immune metabolic pathways contribute to disease pathogenesis. However, the relative contributions of each of these mechanisms to disease pathogenesis are currently unknown. Here we discuss some of these complex pathways, their inter-relationships and their relation to muscle damage in myositis. Understanding the relative contributions of each of these pathways to disease pathogenesis would help us to identify suitable drug targets to alleviate muscle damage and also improve muscle weakness and quality of life for patients suffering from these debilitating muscle diseases.

## Review

Idiopathic inflammatory myopathies (IIMs) include polymyositis (PM), dermatomyositis (DM) and sporadic inclusion body myositis (sIBM). The clinical features of these diseases include muscle weakness, fatigue and elevated muscle enzymes in serum, and their histological characteristics include mononuclear cell infiltration and myofiber degeneration. Immunological features include autoantibodies and autoreactive lymphocytes, with unusual over-expression of major histocompatibility complex (MHC) class I molecules on the surface of the affected myofibers. MHC molecules present processed non-self and self-antigenic peptides to T-lymphocytes and mediate immune response. The relative contribution of the autoimmune component to myositis pathogenesis is not yet known. Recent data suggest that innate immune activation and metabolic defects occur in the myositis muscle, suggesting a role for these pathways in disease pathogenesis [1-3]. Thus, the emerging paradigm indicates that not only innate and adaptive immune mechanisms but also intrinsic defects in skeletal muscle contribute to muscle weakness and damage in myositis. The muscle microenvironment is complex, and we propose that active interactions occur between innate, adaptive, metabolic and homeostatic pathways in muscle in these diseases.

### Innate immune mechanisms

**Figure 1:**
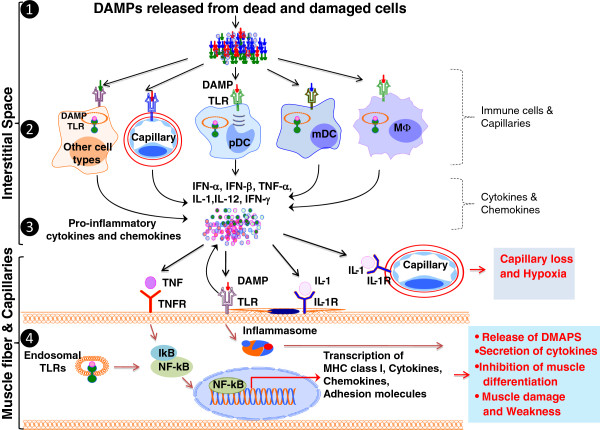
**Innate immune mechanisms of muscle damage in myositis.** Skeletal muscle undergoes continuous injury and repair in response to a variety of physiological (exercise) and pathological (infection) insults and releases damage-associated molecular patterns (DAMPs) from dead and damaged cells (Step **1**). DAMPs initiate innate immune signaling by binding to surface or endogenous TLRs on various cells including skeletal muscle fiber, infiltrating macrophages (Mϕ), myeloid dendritic cells (mDCs), plasmacytoid DCs (pDCs), capillaries, and other cell types such as fibroblasts (Step **2**) [4-6]. This innate signaling through TLR and other innate immune receptors induces the secretion of pro-inflammatory cytokines and chemokines [*e.g.*, Type 1 interferons (IFN-α, IFN-β), TNF-α, IL-1, IL-12 and IFN-γ] into the microenvironment (Step **3**). These cytokines and DAMPs bind to their respective receptors on muscle and capillaries [*e.g.*, tumor necrosis factor receptor (TNFR), IL-1 receptor (IL-1R)] and exert downstream effects (Step **4**) [7-10]. Cytokines and/or chemokines directly cause damage to capillaries and hypoxia in the affected muscle. Cytokines such as TNF-α can directly induce cell death of muscle cells, while NF-kB is known to block MyoD and inhibit formation of the new muscle fibers [11-13]. Thus this pathway not only effectively enhances the death of existing muscle fibers but also inhibits formation of new muscle fibers leading to the loss of skeletal muscle mass and weakness in these disorders.

Innate immunity, also known as native immunity, is considered the early line of host defense. The innate immune system includes physical barriers (epithelial surfaces), phagocytic cells (neutrophils, macrophages, eosinophils, etc.), natural killer (NK) cells, the complement system, and cytokines. Innate immune cells primarily detect pathogen-derived antigen structures with common patterns, but not fine differences, through Toll-like receptors (TLRs) and nucleotide-binding oligomerization domain (NOD)-like receptors (NLRs), to initiate pro-inflammatory responses. We discuss TLRs, NLR-inflammasomes, NF-kB, and cytokines in the context of muscle inflammation below. All the information discussed in this section is summarized in Figure 1.

#### TLR signaling in skeletal muscle

TLRs are the trans-membrane receptors expressed on immune and non-immune cells that recognize pathogens as well as self-molecules. Altogether, 13 TLRs have been identified in mice and humans. All TLRs, except TLR-3, signal via myeloid differentiation response gene 88 (MyD88), the central adaptor protein, and induce activation of the nuclear factor-kB (NF-kB) pathway, the master controller of inflammation. TLR-3 signals via Toll interleukin (IL)-1 receptor domain-containing adaptor inducing IFN-γ (TRIF) and activates the NF-kB pathway or type I interferons (IFNs) [1,2,14]. TLRs recognize patterns in microorganisms termed as pathogen-associated molecular patterns (PAMPs) and endogenous ligands termed as damage associated molecular patterns (DAMPs), and initiate immune signaling [15,16]. PAMPs are associated with infectious agents (*e.g.*, bacteria, fungi and viruses) whereas DAMPs are host-encoded molecules released during tissue injury, necrosis and cell death. DAMPs include nucleic acids (RNA, DNA), cytosolic heat shock proteins and nuclear high mobility group box protein 1 (HMGB1), and extracellular matrix proteins such as fibrinogen and fibronectin [5,6,17]. DAMPs have been shown to induce stimulation of TLRs, resulting in immune activation and the release of cytokines, resulting in a self-sustaining autoinflammatory response that contributes to chronic inflammation in the affected tissue [18-21].

Excessive physical activity and strenuous exercise in normal individuals leads to modest elevations in serum muscle enzymes such as creatine kinase (CK), whereas myositis patients generally show a significant increase in CK, suggesting that skeletal muscle leakiness and damage occur in this disease. It is likely that some DAMPs leak from the injured skeletal muscle and engage their receptors on both skeletal muscle and immune cells, thereby perpetuating the inflammatory process. In fact, muscle biopsies of myositis patients show a significantly increased expression of TLR-2, TLR-3, TLR-4, and TLR-9 in the skeletal muscle and infiltrating cells as well as the enhanced expression of cytokines such as IFN-γ, IL-4, IL-17, TNF-α, IL-6 and type 1 IFNs. These findings suggest that TLR receptors are engaged in the milieu of affected muscle and that the downstream genes are activated [7-9]. Further, IFN-β and IFN-γ are shown to enhance MHC class I expression on immature muscle precursors, suggesting that these cells may be one of the sources of local type 1 IFNs and that the regenerating fibers are potential targets of immune attack in myositis muscle [22].

More recently, one study has independently validated the enhanced expression of TLR-2, -4, and −9 along with MyD88 mRNA transcripts, as well as enhanced protein levels in all subtypes of inflammatory myopathies [10]. The evidence for activation of TLR-4, MyD88, and the NF-κB pathway is also shown in a myosin-induced experimental autoimmune myositis (EAM) mouse model [23]. An enhanced expression of transcripts such as IFN-γ, IL-12p40, and IL-17 along with the expression of the co-stimulatory molecules CD80 and CD86 in the inflammatory milieu of the affected muscle suggests the link between innate and adaptive immune systems in the muscle microenvironment [10].

Recognition of DAMPs that activate the TLR pathway in myositis muscle is slowly emerging. For example, the histidyl-tRNA-synthetase (HRS) protein has long been associated with myositis, since it was identified as the antigen of the myositis-specific autoantibody Jo-1. Previous studies indicated that cleaved HRS serves as a chemokine by binding to CCR5 and facilitates immune cell infiltration into muscle [24]. More recent studies indicate that the N-terminal portion of the HRS protein binds to TLRs, and immunization with HRS peptides induces both autoantibody formation and immunoglobulin class switching in mice. A loss of TLR-4 inhibits class switching, and a loss of TRIF inhibits both class switching and autoantibody secretion [25]. The exact mechanisms by which HRS cleavage and release from muscle cells occurs is unclear, but there is evidence that HRS-expressing immature muscle cells express high levels of MHC class I and therefore likely become targets of cytotoxic T-cells and granzyme B-mediated cleavage of the HRS antigen [26].

Another well-characterized DAMP that is involved in myositis pathogenesis is high mobility group box protein 1 (HMGB1). High expression of HMGB1 was detected not only in the cytoplasm of muscle, infiltrating cells and endothelial cells, but also in the interstitial space in myositis muscle suggesting its potential to engage TLRs in this milieu [4]. Exposure of HMGB1 to muscle fibers induced irreversible decrease in calcium release from the sarcoplasmic reticulum during fatigue induced by repeated tetanic contractions [27]. A recent study reported that HMGB1 induced muscle fatigue occurs via the TLR-4 pathway in muscle and that the HMGB1-TLR-4 pathway plays a role in the pathogenesis of myositis patients [4].

Taken together, these studies clearly suggest that TLRs, acting through MyD88-dependent and/or independent mechanisms, induce pro-inflammatory signals in myopathic muscle. It is likely that new advances in this field would identify additional novel DAMPs in myositis muscle. Blocking DAMP induced MyD88 dependent and independent TLR pathways using chemical and genetic methods may provide additional insights into these mechanisms. Although there are substantial gaps in our knowledge of the relationship between myositis and TLRs, and their stimulation by endogenous DAMPs, the accumulating evidence suggests that the TLRs are the connecting link that mediates interactions between innate and adaptive responses and in turn activates NF-kB signaling cascades in myositis.

#### NF-kB and NLR-inflammasome activation in skeletal muscle

The NF-kB pathway is one of the predominant regulators of a variety of essential biological processes, including inflammation. In myositis both immune and skeletal muscle cells modulate inflammation via the NF-kB pathway. NF-kB is a ubiquitous transcription factor composed of a heterodimer with two subunits, p65 (Rel A)/c-Rel/Rel B and p50. NF-kB is kept sequestered in an inactive form in the cytoplasm through an interaction with its specific inhibitor IkBα. When a stimulus is received, the upstream IkB kinase (IKK) phosphorylates IkBα, leading to its proteosomal degradation. Free NF-kB is then translocated to the nucleus, where it regulates the expression of several pro-inflammatory genes, including TNF-α and IL-1β. We have previously demonstrated that unusual overexpression of MHC class I on the muscle fibers of myositis muscle can also cause the activation of NF-kB, including the induction of ER stress response pathways [27]. Further evidence suggests that downstream NF-kB target genes such as intercellular adhesion molecules (ICAM) and MCP-1 are also highly up-regulated in myositis muscle. Several groups have independently validated NF-kB activation in inflammatory myopathies and its role in modulating the immune response, myogenesis and muscle repair [11-13,28].

NLR-inflammasomes are intracellular multi-protein complexes formed by the adaptor molecule apoptosis-associated speck-like protein with caspase recruiting domain (ASC), caspase-1, and the members of the NLR family such as NLRP1, NLRP3 and NLRC4. NLR-inflammasomes are also activated by PAMPs/DAMPs and result in secretion of the pro-inflammatory cytokines [29,30]. Although the process is not yet completely understood, the general consensus is that inflammasomes are activated through three signaling pathways: 1) potassium efflux, 2) generation of reactive oxygen species, and 3) production of cathepsin B [31]. More recently, our group has shown that normal primary skeletal muscle cells are capable of secreting IL-1β in response to combined treatment with TLR-4 ligand, lipopolysaccharide and P2X7 receptor agonist, ATP, suggesting that not only immune cells but also muscle cells can actively participate in inflammasome formation implicating skeletal muscle cells in perpetuating a pro-inflammatory environment [32].

The inflammasome pathway is connected to the TLR signaling pathway. TLR-2/4 signaling results in the synthesis of pro-IL-1β, and inflammasomes process pro-IL-1β into mature IL-1β; signaling by released extracellular ATP via P2X7 receptors (DAMP signaling) facilitates the secretion of mature IL-1β from the skeletal muscle cells [32]. Another recent study has characterized the mechanism of IL-1β secretion following respiratory syncytial virus (RSV) infection of airways [33]. This study underscored the requirement for the (TLR-2)/MyD88/NF-κB pathway prior to the activation of the inflammasomes and subsequent IL-1β release in the affected tissue [33]. In sum, these findings suggest a possible cross-talk between TLRs and inflammasome pathways. In myositis, the activation of inflammasomes and the subsequent release of cytokines in affected muscle have not yet been investigated; however, enhanced expression of both TLRs and IL-1α and IL-1β in areas surrounded by inflammatory cells suggest that TLR-inflammasome pathway is active in myositis muscle [34]. Therefore, it is possible that the cytokines released from the activation of inflammasome pathways can stimulate innate and adaptive immune cells and further augment the secretion of either pro-inflammatory or anti-inflammatory cytokines.

#### Cytokines and chemokines in skeletal muscle

Cytokines are produced by a wide variety of cells and regulate immune cell activation and infiltration in affected tissues. The most predominantly reported cytokines in myositis include pro-inflammatory cytokines such as IL-1α, IL-1β, TNF-α and transforming growth factor (TGF)-β [34-39]. IL-1α was predominantly expressed in capillary endothelial cells of PM, DM and sIBM muscle biopsies suggesting a prominent role for endothelial cells in myositis pathology [34,35]. Furthermore, IL-1α was suggested to play a role in myofibrillar protein break down and muscle regeneration; however, these claims are yet to be proven [36]. The pathogenic role of TNF-α in myositis muscle was not completely understood; however, it has been hypothesized to attract immune cells by enhancing transendothelial cell trafficking in affected muscle [37]. In addition, TNF-α has been hypothesized to activate immune cells and induce MHC class I expression in the myositis muscle. TGF-β was proposed to play a pro-fibrotic role based on the correlation between its expression and connective tissue proliferation in DM muscle [39]. A plethora of studies have also reported the expression of additional cytokines and chemokines in myopathic tissues [40-50] (Table 1).

**Table 1:** Some of the important cytokines/chemokines reported in inflammatory myopathies

**Cytokines/Chemokines**	**Potential role**	**References**
IL-1α/IL1-β	Pro-inflammatory and probably myofibrillar protein break down	[34-36]
TNF-α	Chemo-attractant	[37]
TGF-β	Pro-fibrotic	[39]
IL-17	IL-6 production and HLA class I in muscle cells	[40,41]
IL-15^1^	T-cell activation, development of NK cells and NK-T-cells	[51]
Type 1 interferons (IFN-α, IFN-β)	Enhance type 1 interferon inducible transcripts (ISG15, MX1, IFIT3 and IRF7)	[42-44]
Leukotriene B4	Chemo-attractant	[45]
Macrophage inflammatory proteins (1α, 1β)	Contribute to ongoing muscle inflammation	[46]
RANTES^2^	Chemo-attractant	[46]
Resistin/Adipocyte secreted factor	Pro-inflammatory, probably involved in metabolic dysregulation	[47-49]
TWEAK^3^	Impairs muscle differentiation and myogenesis	[50]

^1^IL-15 and IL-6 are also called myokines.

^2^RANTES: Regulated on activation, normally T expressed and secreted.

^3^TWEAK: Tumor necrosis factor like weak inducer of apoptosis.

Even though a majority of the reports suggest that cytokines have a pro-inflammatory role in myositis muscle, one recent study reported a protective role for some cytokines. This study reported enhanced expression of neurotrophin receptor p75NTR on the muscle fibers of DM, PM and sIBM patients [52]. p75NTR binds to various neurotrophin-like cytokines such as NGF, BDNF, NTF3 or NTF4, and protects muscle cells against IL-1β induced cell death. Taken together, these studies indicate that cytokines and chemokines have different roles in the affected skeletal muscle.

### Adaptive immune mechanisms

**Figure 2:**
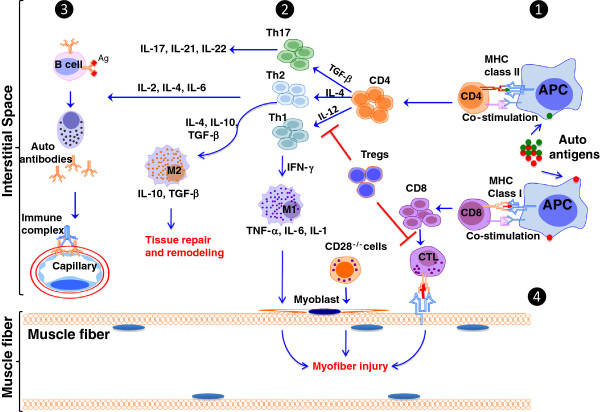
**Adaptive immune mechanisms of muscle damage in myositis.** DAMP signaling through TLRs in the innate immune cells activates various antigen-presenting cells (APC) in the muscle (shown in Figure 1). These APCs activate CD4 T-cells via MHC class I and CD8 T-cells initiate autoantigen specific T-cell responses (Step **1**) [26]. Activated CD4+ T-cells differentiate into T-helper (Th)-17 (TGF-β), Th2 (IL-4), and Th1(IL-12) effector T-cells in the presence of respective cytokines, and in turn produce discrete sets of cytokines that affect a variety of cell types (Step **2**) [53]. Th1 cells through IFN-γ generate M1 macrophages, which secrete TNF-α, IL-6 and IL-1, and damage cells. Th2 cells, through IL-4, TGFβ and IL-10, generate M2 macrophages that are known to help tissue repair and remodeling in the affected tissues [54,55]. Th2 cells also help stimulate B-cell maturation and differentiation into plasma cells that produce autoantibodies and further initiate complement mediated damage to capillaries and induce hypoxia (Step **3**). Cytotoxic CD28^−/−^ T-cells and regulatory T-cells (Tregs) reduce inflammation and tissue damage by inhibiting the function of antigen presenting cells and T-effector cells [56,57]. It is also known that activated CD8 T-cells differentiate into cytotoxic T-cells (CTL) and exert cytotoxic effects on the affected muscle through secretion of perforin-1 and granzyme-B enzymes (Step **4**) [58]. Thus the myositis muscle microenvironment is complex, with both tissue repair and tissue-damaging mechanisms in play at all times. The relative ratios of these pathways determine the disease severity and progression.

Adaptive immunity to self-antigens is induced in autoimmune diseases. This arm of immunity predominantly includes autoreactive lymphocytes and autoantibodies. Initial reports have indicated that there are differences in the lymphocyte subsets seen in PM, DM and sIBM; however, recent studies have indicated that those differences are not clear-cut and that T-cells (CD4, CD8), B-cells, macrophages, and DCs are present in all inflammatory myopathies. All the information discussed in this section is summarized in Figure 2.

#### T-cells and CTL-cell-mediated injury

T-cells are involved in cell-mediated immune responses within the adaptive immune system. These cells express surface receptors (T-cell receptors; TCR) that recognize peptide fragments of foreign proteins when presented on the MHC molecules of antigen-presenting cells. Functional subsets of T-cells include CD4+ T helper cells (which recognize MHC class II-presenting peptides) and CD8+ cytotoxic T-cells (which recognize MHC class I-presenting peptides). The role of CD4+ and CD8+ T-cells in inflammatory myopathies has been recognized; however, their precise roles in the pathogenesis of myositis are not completely understood. In the pathology of DM, CD4+ T-cells are thought to play a major role; in contrast, CD8+ T-cells seem to be the predominant actors in PM [59,60]. CD8+ T-cells infiltrating myositis muscle have been shown to express perforin-1 and granzyme-B enzymes, indicating that they have a cytotoxic effect on the affected muscle (Figure 2) [58]. Recent studies demonstrate the presence of CD28^null^ T-cells, Th17 cells, and T-regulatory cells in the muscle of PM and DM patients [53,56,57] (Figure 2). The CD28^null^ T-cells arise as a result of a chronic inflammatory stimulus (such as infection from virus) and are generally long-lived and pro-inflammatory in nature. Likewise Th17 cells produce IL-17 and IL-22. IL-22 has both tissue protection and pro-inflammatory properties. Contribution of Th17 cells to inflammatory process in autoimmune diseases, such as rheumatoid arthritis, is well delineated. Regulatory T-cells, which express CD25, reduce inflammation and tissue damage by inhibiting the function of antigen presenting cells and T-effector cells. Even though the presence of different T-cell subpopulations in myositis muscle has been well documented, their precise role in muscle pathology is not yet clear.

#### B-cells and autoantibodies

B-cells that are derived from bone marrow migrate to secondary lymphoid organs to elicit antigen specific humoral immune response. B-cells and terminally differentiated plasma cells have also been reported not only in PM and DM but also in sIBM, indicating their role in the pathogenesis of these diseases [61]. More recent reports indicating an up-regulation of B-cell activating factor (BAFF) have also suggested that a local maturation of B-cells to antibody-producing plasma cells may occur in myositis muscle [61,62]. Despite the presence of lymphoid aggregates, it is highly unlikely that B-cell maturation occurs in the muscle; rather, these B-cells may serve an antigen-presenting function.

Presence of myositis-specific antibodies against autoantigens such as histidyl-tRNA synthetase (anti-Jo-1) and chromodomain-helicase DNA-binding proteins (anti-Mi-2) has been well established in myositis patients; more than half of all patients show autoantibodies. Several different autoantibodies have been reported in different myopathies [3,63-81] (Table 2). The majority of antibodies reported are directed against ubiquitous cytoplasmic or nuclear components involved in critical cellular regulatory processes and the role of autoantibodies in mediating muscle damage and injury is uncertain in myositis. However, autoantibodies are extremely useful for diagnosing and classifying myositis patients and for predicting disease course and therapeutic outcomes. For more information on myositis autoantibodies, readers are advised to consult the reviews [82,83].

**Table 2:** Some of the important autoantibodies reported in inflammatory myopathies

**Autoantibodies**	**Disease**	**Association**	**References**
Anti-tRNA synthetases^1^ (Anti-Jo; against histidyl tRNA synthetase)	More common in PM than DM	Interstitial lung disease	[63-65]
Anti-chromodomain helicase DNA binding proteins (anti-Mi2)	DM	Cutaneous lesions	[3,66,67]
Anti-MDA5/Anti-CADM-140	DM	Mucocutaneous lesions; severe lung disease minimal muscle involvement	[68-70]
Anti-TIF1γ^2^	DM	Malignancy	[71-73]
Anti-nuclear matrix protein (NXP)-2/anti-MJ	Mostly juvenile DM	Joint contractures; calcinosis	[74]
Anti-SAE^3^	DM	Skin and muscle manifestations	[75]
Anti-signal recognition particle	NM, PM	Degenerating and regenerating muscle fibers and possible cardiac involvement	[76-79]
Anti-HMG-CoA reductase^4^	Statin associated myopathy	Treatment with cholesterol lowering drugs	[80,81]

*PM* Polymyositis, *DM* Dermatomyositis, *NM* Necrotizing myopathy.

^1^Additional antisynthetase antibodies found in myositis are targeted against threonyl-tRNA synthetase (PL-7); alanyl-tRNA synthetase (PL-12); isoleucyl-tRNA synthetase (OJ); glycyl-tRNA synthetase (EJ); asparaginyl-tRNA synthetase (KS).

^2^TIF1γ: Transcription intermediary factor 1γ.

^3^SAE: Small ubiquitin like modifier activating enzyme.

^4^HMG-CoA reductase: 3-hydroxy-3-methylglutaryl-coenzymeA reductase.

#### Dendritic cells connect the innate and adaptive arms of the immune system

There is clear evidence that innate and adaptive immune cytokines influence each other. For instance, IL-18 stimulates the secretion of IFN-γ and TNF-α via a Th1-mediated response [84,85]. Similarly, IL-1β binds to IL-1 receptor on dendritic cells and produces IL-23 via a Th17-mediated response, and IL-33 binds to IL-1 receptor-related protein (ST2) and enhances the secretion of IL-10 and IL-13 through Th2-mediated responses [86]. IL-33 also induces the secretion of IL-13, IL-10 and TGF-β by stimulating mast cells and T-reg cells [86]. These interactions through cytokines highlight that innate and adaptive immune processes are interrelated and studies to understand their role in muscle disease pathogenesis are imminent.

DCs are bone marrow-derived immune cells that connect innate and adaptive immune systems. DCs are considered professional antigen-presenting cells, and their main function is to prime and activate naïve T-lymphocytes. Immature DCs express CD1a and blood dendritic cell antigen 2 (BDCA2) surface markers, whereas mature DCs express DC-LAMP, CD83 and fascin surface markers. We have previously shown that DC-LAMP-positive dendritic cells are highly enriched in perivascular inflammatory sites in juvenile and adult DM patients, along with molecules that facilitate dendritic cell transmigration and reverse transmigration (CD142 and CD31) [87]. Both immature and mature DCs have been found to be present in DM and PM biopsies [88,89]. Recent studies have reported that myeloid DCs may regulate type I IFN-mediated induction of cytokines and chemokines in DM muscle, indicating an association between DCs and type I IFN signatures in myositis muscle [90]. More recently, plasmacytoid DCs (pDCs) have also been implicated in myositis pathology. pDCs are innate immune cells with a plasma-cell morphology that express CD4 or the myeloid-cell markers MHC class II, CD36, CD68 and CD123 [91]. pDCs characteristically produce type I IFNs and other chemokines in response to virus-derived nucleic acids, via the activation of endosomal TLR-7 and TLR-9 pathways (Figure 1). They may serve as an essential link between innate and adaptive immune mechanisms through the secretion of type 1 IFNs and other cytokines [92,93].

Macrophages are tissue-based phagocytic cells derived from peripheral monocytes. They carry out a multitude of functions, including antigen presentation to T-cells and scavenging of necrotic tissues via phagocytosis. Different types of macrophages in the muscle clearly influence the type of the adaptive immune response (*e.g.*, Th1 or Th2). Distinct subpopulations of macrophages have been described; M1 macrophages, in association with Th1 cells, produce pro-inflammatory mediators and are involved in the phagocytosis of microorganisms and neoplastic cells. M2 macrophages are Th2-associated and are involved in tissue remodeling/repair and the production of anti-inflammatory molecules. Depending on their stage of activation, macrophages exhibit different surface markers; MIF-related protein (MRP) 14 and 27E10 represent early-stage markers; 25F9 is a late-activation marker. Infiltration of macrophages into myositis tissues and the presence of CD163 positive (M1) macrophages are described in myositis muscle [4,54,55]. Characterization of macrophage subtypes in PM and DM muscle indicated that they express both early, MRP14 and 27E10 (M1 macrophage) and late activation 25F9 (M2 macrophage) and inflammatory markers such as iNOS and TGF-β [54,55]. These studies indicate that both M1 and M2 macrophages exist in the myositis muscle and their relative proportions may vary depending on the stage of the disease process. Therefore, interactions between innate immune cells/cytokines and lymphocytes appear to be dynamic and alter with the type and stage of the disease.

### Non-immune mechanisms

**Figure 3:**
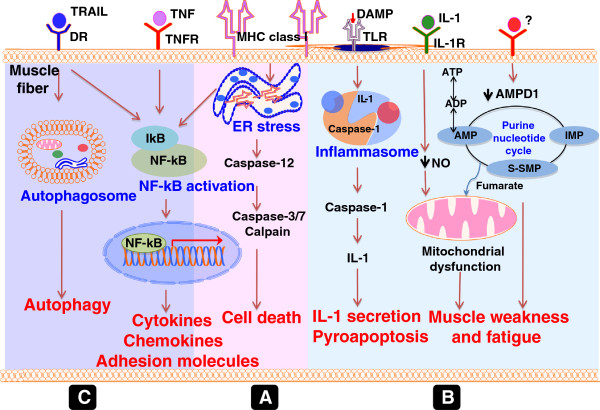
**Non-immune mechanisms of muscle damage and weakness.** MHC class I overexpression on myofibers make muscle susceptible for CD8 T-cell mediated cytotoxicity as well as susceptible to endoplasmic reticulum stress-induced cell death. MHC class I accumulation in endoplasmic reticulum induces stress responses (unfolded protein response and endoplasmic reticulum overload response (EOR)) [27,94-98]. Induction of EOR activates downstream NF-kB pathway leading to pro-inflammatory cytokine production and reduction in new muscle formation by inhibiting MyoD. It also induces cell death mechanisms via the activation of caspases 12, 3 and 7 as well as calpain pathways (Step **A**) [27]. Innate cytokines, mitochondrial energy-related metabolic pathways, and purine nucleotide pathways are interconnected in myositis muscle. For instance, IL-1 reduces the production of nitric oxide (NO) and causes mitochondrial dysfunction by affecting NADH reductase and succinate CoQ [99-102]. Likewise, unknown cytokines reduce expression of rate-limiting enzymes of the purine nucleotide cycle and of AMPD1 in skeletal muscle. This acquired deficiency of APMD1 causes muscle weakness and fatigue in myositis (Step **B**) [103]. Activation of TRAIL forms autophagosomes and induces autophagy (Step **C**) [104]. TLR signaling leads to inflammasome activation, IL-1 secretion and pyroapoptosis in the affected muscle. There are active interactions between autophagy, ER stress, and inflammasome and purine nucleotide pathways. Even though all these pathways are interconnected, we have represented them as linear pathways in this illustration for easier understanding. Thus, several non-immune and metabolic pathways directly and indirectly contribute to muscle weakness and damage in myositis.

Because of the presence of immune cells, it is generally thought that myofiber damage is the consequence of an immune process to muscle derived antigen. However, several observations suggest the involvement of non-immune mechanisms in myositis pathology: 1) the lack of a correlation between the degree of inflammation and skeletal muscle weakness; 2) the lack of a response to potent immunosuppresants by some myositis patients; and 3) the lack of any amelioration of clinical disease even after complete removal of inflammatory infiltrates from the myositis muscle. Here we describe the literature related to skeletal muscle homeostasis and metabolism that supports a role for non-immune mechanisms in myositis pathology. Hereditary IBM (hIBM) is a an autosomal recessive muscle disorder tied to a mutation in the UDP-N-acetylglucosamine 2-epimerase/N-acetylmannosamine kinase (GNE) that codes for a rate-limiting enzyme in the sialic acid biosynthetic pathway. Pathogenesis of hIBM is considered non-inflammatory and is not discussed in this review. All the information discussed in this section is summarized in Figure 3.

#### Metabolic/energy pathways in skeletal muscle

Mitochondrial energy-related metabolic pathways play a prominent role in skeletal muscle because of the high demand for energy in these cells. Mitochondria can regulate various signaling pathways via the production of ATP, NADH and reactive oxygen species. Emerging evidence indicates a probable dysregulation of mitochondrial energy pathways in inflammatory muscle diseases [99,105]. Studies have reported abnormal succinic dehydrogenase and cytochrome c oxidase (COX) activities in DM muscle and observed that these abnormalities are more pronounced in damaged, atrophic perifascicular fibers [100,101]. Pro-inflammatory cytokines (specifically TNF-α) have also been shown to affect muscle metabolism, leading to weakness. TNF-α acts via the TNFR1 receptor subtype and reduces the specific force generated by muscles. This reduction in force is attributed to increased cytosolic oxidant activity and decreased myofibrillar function and specific force without altering calcium regulation or other aspects of myofibrillar mechanics [102]. These findings indicate a potentially detrimental effect of pro-inflammatory cytokines on skeletal muscle and mitochondrial energy metabolic pathways.

One of the often-overlooked features of myositis is the apparent acquisition of metabolic defects within the skeletal muscle. These defects are generally described as deficiencies of glycolytic enzymes and other proteins found preferentially in fast-twitch fibers. One of the oldest proposed metabolic defects in inflammatory myopathies is an acquired deficiency of a rate-limiting enzyme, AMPD1, in purine nucleotide cycle [106,107]. Recently, our group demonstrated that AMPD1 mRNA, protein expression and enzyme activity are significantly reduced in the MHC class I mouse model of myositis, as compared to healthy littermate mice [103]. A cause-and-effect relationship between AMPD1 and muscle weakness has been demonstrated by reducing the levels of AMPD1 in normal mice. The most novel observation was that a significant loss of AMPD1 enzyme activity and muscle strength occurs prior to the appearance of infiltrating lymphocytes. These results suggest that the metabolic deficiencies seen in myositis are independent of the action of infiltrating autoreactive lymphocytes.

At this time, it is unclear what factors/cytokines regulate AMPD1 levels in skeletal muscle. Evaluation of the AMPD1 promoter has indicated that cytokines are likely to modulate AMPD1 expression in skeletal muscle. For example, the cytokine IL-15 has the potential to serve as a link between inflammation and muscle metabolism. IL-15 was first described as a weak ligand for the IL-2 receptor complex, and as such is capable of stimulating T-cell proliferation, among other immunomodulatory effects. Recent work has shown that IL-15 signaling affects the formation of fast-twitch fibers in mice; in the absence of the IL-15 receptor, muscle fibers appear to convert from fast-twitch to slow-twitch fibers [108]. Furthermore, strong staining for IL-15 has been detected in myoblasts but not in mature muscle fibers [51]. These results are particularly interesting, considering the previously mentioned evidence that immature fibers may become a focal point of inflammation as a result of the secretion of IL-15, and the subsequent loss of these IL-15-positive fibers might explain the observed shift toward slow-twitch fibers in myositis patients [51]. Even though the precise role of these metabolic pathways in the myofiber damage seen in myositis is not yet clear, it is possible that innate TLR pathways and pro-inflammatory cytokines regulate these mechanisms.

#### Endoplasmic reticulum stress

A non-immune role for MHC class I has been reported in myositis. Muscle-specific overexpression of MHC class I causes the myositis phenotype in mouse skeletal muscle [109]. Studies have reported an induction of endoplasmic reticulum stress as the result of an unusual up-regulation of MHC class I in myositis muscle [27,94-96]. More recently, studies to understand the role of endoplasmic reticulum stress in muscle pathology reported the expression of classical markers of endoplasmic reticulum stress (GRP78, GRP94 and calreticulin) in the affected skeletal muscle of both mice and humans [27,97,98,110]. A recent study has reported the presence of stress response proteins and heat shock proteins (Hsp) in IIM patients [111]. More specifically, the authors have examined the effects of chronic inflammation on the distribution of Hsp families 70 and 90 in muscle biopsies. Their results have indicated that regenerating, atrophic and vacuolated muscle fibers show an upregulation of both protein families, whereas infiltrating cells show enhanced levels of Hsp 90 family proteins. These results indicate a differential expression of stress proteins in muscle cells and immune cells. Thus, the authors suggest that chaperones play multifaceted roles in inflammatory muscle tissue. For more detail and a comprehensive discussion of the relationship between endoplasmic reticulum stress and muscle pathology, readers are referred to a recent review on this subject [112].

#### Autophagy

Autophagy is the lysosomal degradation of a cell’s own proteins or organelles. Evidence of autophagy is often seen in PM and sIBM. Muscle biopsies from humans with sIBM and PM with mitochondrial pathology display the autophagosome marker LC3-II [99]. However, the precise role of autophagy in muscle diseases is controversial. It is likely that autophagy has both beneficial and adverse effects, depending on the cell stage and disease process involved. The *in vitro* inhibition of lysosomal autophagic enzymes has been reported to activate γ-secretase, which cleaves amyloid precursor protein to release the self-aggregating amyloid-β fragment [113]. We have demonstrated that TNF-related apoptosis-inducing ligand (TRAIL) and markers of autophagy are up-regulated in myositis muscle fibers. Incubation of skeletal muscle cells with TRAIL induces IκB degradation and NF-κB activation, suggesting that it mediates the activation of NF-κB as well as autophagic cell death in myopathic muscle [104]. Another recent report has also indicated that TNF-α induces macroautophagy and subsequent expression of MHC class II on muscle cells [114]. More importantly, blockade of TNF-α with monoclonal antibodies has been shown to improve C protein-induced myositis (CIM) in mice, suggesting a probable role for autophagic pathways in myositis pathology [115]. In addition, immunomodulators such as fibrinogen and HMGB1 are correlated with the progression of myositis and are believed to induce autophagy by signaling through TLR-4, indicating a probable association with innate immune mechanisms [116]. Even though these findings indicate that autophagy plays a role in myofiber damage in myositis, further studies are needed to show how and when these autophagic mechanisms are triggered in the affected muscle.

## Conclusions

The emerging picture indicates that myositis is a complex disease with multiple pathogenic pathways simultaneously contributing to muscle damage and weakness. Among these, the most prominent are the innate, adaptive immune and metabolic pathways. Innate immune pathways link the adaptive and metabolic arms of the disease processes. Additional new pathways and the precise interactions between these components are likely to be described in the future, and the relative contribution of each of these pathways to pathogenesis remains to be elucidated. However, it is clear that targeting the adaptive immune system alone is unlikely to provide significant relief from muscle weakness and damage in this group of disorders. New therapies are needed to modulate both the innate immune and metabolic components of the disease processes in order to obtain significant amelioration of the myositis phenotype.

## Abbreviations

AMPD1: Adenosine monophosphate deaminase 1; ASC: Apoptosis-associated speck-like protein with caspase recruiting domain; BDCA2: Blood dendritic cell antigen 2; CIM: C protein-induced myositis; CK: Creatine kinase; COX: Cytochrome c oxidase; DAMP: Damage-associated molecular pattern; DC: Dendritic cells; DM: Dermatomyositis; EAM: Experimental autoimmune myositis; hIBM: Hereditary inclusion body myositis; HMGB1: High mobility group box protein 1; HRS: Histidyl-tRNA-synthetase; Hsp: Heat shock protein; ICAM: Intercellular adhesion molecules; IFN: Interferon; IIM: Idiopathic inflammatory myopathy; IKK: IkB kinase; IL: Interleukin; MHC: Major histocompatibility complex; MyD88: Myeloid differentiation response gene 88; NF-kB: nuclear factor-kB; NK: natural killer; NLR: Nucleotide-binding oligomerization domain (NOD)-like receptor; PAMP: Pathogen-associated molecular pattern; PM: Polymyositis; sIBM: Sporadic Inclusion body myositis; TGF: Transforming growth factor; TLR: Toll-like receptors; TNF: Tumor necrosis factor; TRAIL: TNF-related apoptosis-inducing ligand; TRIF: Toll-interleukin receptor domain-containing adapter-inducing interferon-β.

## Competing interests

The authors declare that they have no competing interests.

## Authors’ contributions

SR and KN were involved in drafting all sections of the manuscript and revising it critically for important intellectual content. WC and TBK were involved in writing non-immune mechanisms section. All authors read and approved the final manuscript.
